# Stress fracture of the neck of talus in an adolescent female with hypothyroidism: a case report of an unusual cause of ankle pain

**DOI:** 10.1097/RC9.0000000000000430

**Published:** 2026-03-25

**Authors:** Bimal Rai, Vinayak Dhungana, Deekshya Devkota

**Affiliations:** a Department of Orthopedic Surgery, Shree Birendra Hospital, Kathmandu, Nepal; b Nepalese Army Institute of Health Sciences, Kathmandu, Nepal

**Keywords:** case report, hypothyroidism, levothyroxine, military, stress fracture, talus neck

## Abstract

**Introduction:**

Stress fractures occur when repetitive, cyclic loading exceeds the bone’s capacity for remodeling. Talar stress fractures are rare, typically affecting the head or body, while involvement of the talar neck remains largely undocumented. Early diagnosis is essential, as even high-risk fractures can be managed successfully with conservative treatment.

**Case presentation:**

An 18-year-old hypothyroid female on levothyroxine therapy (37.5 mcg/day), undergoing intense training for military recruitment, presented with persistent anteromedial right ankle pain for 5 months. Examination revealed an antalgic gait and hindfoot tenderness without restriction in ankle motion. While initial radiographs revealed normal findings, magnetic resonance imaging (MRI) confirmed a nondisplaced stress fracture at the talar neck (Kaeding–Miller Grade II). The patient was treated conservatively with 6 weeks of non-weight-bearing immobilization, nonsteroidal anti-inflammatory drugs (NSAIDs), and structured rehabilitation, resulting in complete recovery and return to full activity.

**Discussion:**

Talar neck stress fractures are exceptionally rare and pose diagnostic challenges due to non-specific symptoms and often inconclusive radiographs. This case emphasizes the importance of considering metabolic contributors – particularly thyroid dysfunction and levothyroxine therapy – as potential risk factors for stress injury in active females. Early MRI was critical in confirming the diagnosis and guiding appropriate management.

**Conclusion:**

This case demonstrates that early identification of a talar neck stress fracture using MRI, combined with prompt conservative management, can lead to full recovery even in high-risk fracture locations. Clinicians should maintain a high index of suspicion in active patients with metabolic risk factors and persistent ankle pain.

## Introduction

Stress fractures generally occur due to repetitive, cyclic loading that exceeds the mechanical capacity of the bone^[^1^]^. Kaeding and Miller suggested a classification system for stress fractures based on clinical presentation and radiographic evidence^[^2^]^. Stress fractures of the talus are relatively rare, with an incidence of only 4.4 per 10 000 cases in military recruits, and most commonly occur in the talar head and body^[^3^]^. McGlone first reported stress fractures in the talus in 1965^[^4^]^. There have been several case reports of the talar body^[^5–7^]^, but the literature regarding stress fractures of the talar neck is sparse, posing diagnostic and therapeutic challenges.

We present a case of an 18-year-old hypothyroid female, training for military recruitment, who developed a stress fracture of the talar neck after intense military training. This case report adheres to SCARE 2025 guidelines^[^8^]^ and addresses a gap in the current literature by presenting the clinical presentation, radiographic findings, conservative management, rehabilitation strategy, and outcomes of a Kaeding and Miller type II talar neck stress fracture.

**HIGHLIGHTS**

- Talar neck stress fractures, although rare, should be considered in the differential diagnosis of persistent ankle pain in active individuals, particularly those undergoing high-impact training.
- This case report emphasizes the need to consider thyroid dysfunction and levothyroxine therapy as potential contributors to rare stress fractures in young female athletes.
- Early use of advanced imaging, such as MRI, is essential in diagnosing stress fractures, as initial plain radiographs may be inconclusive.
- Even high-risk stress fractures can be successfully managed with timely non-operative treatment, including immobilization, structured rehabilitation, and gradual return to activity.

## Case presentation

An 18-year-old female training for military recruitment presented with persistent pain in the anteromedial region of her right ankle for 5 months. The pain developed gradually during intensified physical training and running sessions and was 7/10 in the numeric pain rating scale. Before military training, she had no history of ankle pain and was accustomed to running less than a mile. However, after starting her recruitment program – which involved running around 6 miles daily – she started experiencing aching pain that occasionally radiated to her midfoot. The pain worsened with weight-bearing activities, especially running and downhill walking, but improved with rest. She also noted swelling and tenderness in the affected area, although there was no history of acute trauma.

She was a known case of hypothyroidism, for which she was under medication (levothyroxine 37.5 mcg once daily) and was euthyroid on presentation. Her thyroid-stimulating hormone (TSH) was 0.61 mIU/L (reference range 0.38–5.33), free T3 was 3.38 pg/mL (reference range 2.5–3.9), and free T4 was 1.17 ng/dL (reference range 0.58–1.64) at presentation. The patient had no menstrual irregularities or abnormalities. Her past history includes a conservatively treated fracture at the base of the fifth metatarsal and a laparoscopic cystectomy for a dermoid cyst 5 years earlier.

On examination, her general condition was fair, and vital parameters were within normal limits. However, she exhibited an antalgic gait during weight-bearing and was unable to walk downstairs due to pain. Local examination revealed tenderness over the hindfoot, although active and passive ankle dorsiflexion, plantarflexion, inversion, and eversion were normal, indicating no significant joint motion restriction.

**Figure 1.**
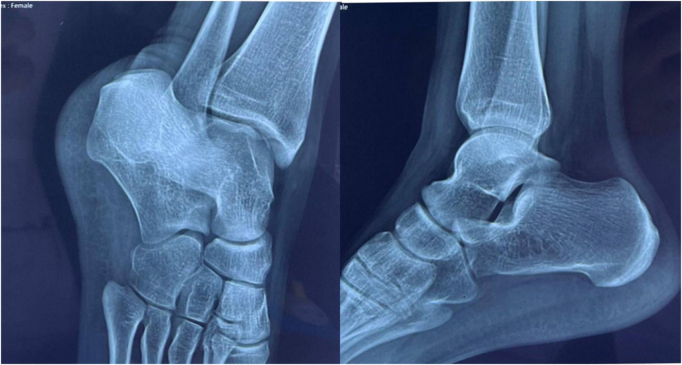
Plain X-ray of ankle, Canale, and lateral view showing normal findings.

**Figure 2.**
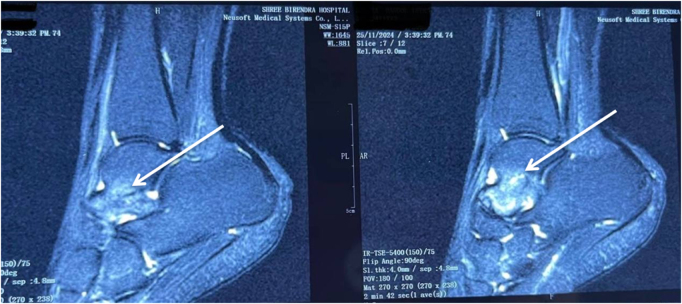
T2-weighted sagittal MRI image showing diffuse hyperintensity at the head and neck region of the talus (white arrows).

Initial plain radiography of the ankle was normal (Fig. 1), while subsequent MRI revealed bone marrow edema and a nondisplaced stress fracture at the talar neck (Fig. 2). These findings, in conjunction with the patient’s clinical history – the absence of acute trauma and insidious onset of pain during intensified training – confirmed the diagnosis of a talar stress fracture (Kaeding–Miller Grade II). The imaging findings and clinical presentation helped distinguish this injury from alternative diagnoses, such as ankle sprains, sinus tarsi syndrome, tarsal coalition, or osteochondral lesion.

The management plan was conservative and involved 6 weeks of non-weight bearing and immobilization, during which nonsteroidal anti-inflammatory drugs (NSAIDs) were prescribed for pain control. The patient was also advised to stop running and high-impact activities for 8 weeks. Following immobilization, rehabilitation focused on gradual weight-bearing, ankle range-of-motion exercises (dorsiflexion, plantarflexion, inversion, and eversion), towel curl and marble pick-up exercises for intrinsic foot muscles, heel-raise exercises, and single-leg balance training along with proprioceptive physiotherapy. After 6 weeks of conservative treatment and 2 weeks of rehabilitation, the patient successfully transitioned to weight-bearing activities with significant improvement in pain (2/10 in the numeric pain rating scale) and was back to her regular activities.

## Discussion

Stress fractures, also known as fatigue fractures, frequently occur in athletes and females when repetitive abnormal stresses on normal bone cause localized cortical resorption and eventual fracture^[^9^]^. Approximately 90% of cases involve the lower extremities, particularly the tibia, tarsal navicular, metatarsals, and femur^[^10^]^. In young females, especially those engaged in rigorous physical regimens like military training or long-distance running, the interplay of thyroid-related hormonal changes and increased mechanical loading can exacerbate the risk of stress fractures^[^11^]^.

Our patient, a young female with hypothyroidism on levothyroxine therapy (37.5 mcg/day), presented with a non-displaced stress fracture of the talar neck – an exceedingly rare site. While stress fractures typically show good prognosis, high-risk sites like the femoral neck, patella, talus, and medial malleolus are more susceptible to complications such as delayed union, non-union, or re-fracture and may require surgical intervention^[^12^]^.

Among talar stress fractures, the head is most frequently affected, followed by the body, while the posterior talus is rarely involved^[^13^]^. No documented cases of stress fractures in the talar neck have been previously reported. In a case series by Sormaala *et al*, all nine patients with talar stress fractures (eight involving the head and one the body) were successfully managed conservatively without cast immobilization^[^14^]^. Similarly, Manzotti *et al* described a talar head stress fracture in a 24-year-old runner with talocalcaneal coalition, treated conservatively with NSAIDs and proprioceptive rehabilitation^[^15^]^. Motto *et al* reported a lateral talar process fracture in a 52-year-old tennis player managed surgically^[^16^]^.

In contrast to previous cases, our patient presented with a unique stress fracture of the talar neck (Kaeding and Miller type II) and was managed successfully with non-weight bearing, immobilization, analgesia, and structured rehabilitation. This highlights the importance of considering talar neck stress fractures in the differential diagnosis of persistent ankle pain in athletes, particularly those with metabolic risk factors like thyroid disorders.

Recent studies suggest a significant link between thyroid dysfunction and bone fragility. According to Apostu *et al*, both hyperthyroidism and hypothyroidism are associated with decreased bone mineral density (BMD) and increased fracture risk^[^6,17^]^. Thyroid hormones influence both osteoblast and osteoclast activity via the thyroid hormone receptor alpha receptor and the TSH receptor pathways. Hypothyroidism slows bone remodeling and may paradoxically increase bone stiffness, leading to fragility despite normal or elevated BMD readings^[^6,17^]^.

The condition is further complicated by levothyroxine therapy. While essential for correcting hypothyroidism, overtreatment – especially in the lower TSH range – can mimic the effects of hyperthyroidism, increasing bone turnover and reducing BMD^[^6,17^]^. Although our patient was on a relatively low dose, this cannot completely rule out its contribution to bone vulnerability.

A recent systematic review and meta-analysis by Li *et al*^[^18^]^ further emphasize the nuanced impact of levothyroxine on skeletal health in overt hypothyroidism. The study found a statistically significant reduction in lumbar spine BMD in overt hypothyroid patients undergoing levothyroxine treatment compared to healthy controls (SMD: −0.28; 95% CI: −0.55 to −0.02; *P* = 0.04) . Furthermore, Li *et al* reported a trend toward increased osteocalcin and alkaline phosphatase levels in levothyroxine-treated patients, indicating an elevation in bone turnover, even if not statistically significant^[^18^]^. This subtle shift in bone metabolism could be clinically meaningful in patients with concurrent mechanical stress, such as athletes.

Paoletta *et al*^[^19^]^ explored a case of transient osteoporosis of the hip associated with subclinical hypothyroidism, proposing that even subclinical thyroid dysfunction could initiate a cascade of bone marrow changes via altered vascularity, local inflammation, and increased regional turnover. Although their case did not involve mechanical overloading, the metabolic parallels support a multifactorial etiology for bone fragility in thyroid-affected patients.

Thus, our case adds to the limited literature linking hypothyroidism and its treatment to rare stress fractures in young female athletes. It emphasizes the importance of considering endocrine factors such as thyroid function – even when well managed – in patients presenting with stress injuries at uncommon sites. Moreover, it highlights the potential for levothyroxine, even at low doses, to contribute subtly to altered bone integrity, especially in the early phases of therapy. However, a complete metabolic panel was not performed, and imaging was not conducted at the 6-week follow-up, which is a limitation of this case report.

## Conclusion

This rare case of a talar neck stress fracture emphasizes the importance of considering such fractures in the differential diagnosis of persistent ankle pain in active individuals, particularly those engaged in high-impact activities. Early advanced imaging, especially MRI, combined with a comprehensive metabolic evaluation, is crucial for prompt diagnosis and identification of underlying risk factors. Even high-risk stress fractures can achieve successful outcomes with timely non-operative management, including immobilization, structured rehabilitation, and gradual return to activity.

## References

[1] Kaeding CC Miller T. The comprehensive description of stress fractures: a new classification system. J Bone Joint Surg 2013;95:1214–20. 23824390 10.2106/JBJS.L.00890

[2] Kaeding CC Najarian RG. Stress fractures: classification and management. Phys Sportsmed 2010;38:45–54. 10.3810/psm.2010.10.1807 20959695

[3] Sormaala MJ Niva MH Kiuru MJ. Bone stress injuries of the talus in military recruits. Bone 2006;39:199–204. 16466974 10.1016/j.bone.2005.12.001

[4] McGlone JJ. Stress fractures of the talus. J Am Podiatry Assoc 1965;55:814–17. 5846443 10.7547/87507315-55-12-814

[5] Bradshaw C Khan K Brukner P. Stress fracture of the body of the talus in athletes demonstrated with computed tomography. Clin J Sport Med 1996;6:48–51. 8925366 10.1097/00042752-199601000-00010

[6] Rossi F Dragoni S. Talar body fatigue stress fractures: three cases observed in elite female gymnasts. Skeletal Radiol 2005;34:389–94. 15889248 10.1007/s00256-005-0913-z

[7] Kim YS Lee HM Kim JP. Fatigue stress fracture of the talar body: a common cause of ankle pain. J Foot Ankle Surg 2016;55:1113–16. 26961416 10.1053/j.jfas.2016.01.042

[8] Kerwan A Al-Jabir A Mathew G. Revised Surgical CAse REport (SCARE) guideline: an update for the age of Artificial Intelligence. Prem J Sci 2025;10:100079.

[9] Matcuk GR Mahanty SR Skalski MR. Stress fractures: pathophysiology, clinical presentation, imaging features, and treatment options. Emerg Radiol 2016;23:365–75. 27002328 10.1007/s10140-016-1390-5

[10] Kahanov L Eberman L Games K. Diagnosis, treatment, and rehabilitation of stress fractures in the lower extremity in runners. Open Access J Sports Med 2015;6:87–95. 25848327 10.2147/OAJSM.S39512 PMC4384749

[11] Maccagnano G Notarnicola A Pesce V. The prevalence of fragility fractures in a population of a region of southern Italy affected by thyroid disorders. Biomed Res Int 2016;2016:1–6. 10.1155/2016/6017165 PMC5078635 27807539

[12] McInnis KC Ramey LN. High-risk stress fractures: diagnosis and management. Pm&r 2016;8:S113–24. 26972260 10.1016/j.pmrj.2015.09.019

[13] Welck M Hayes T Pastides P. Stress fractures of the foot and ankle. Injury 2017;48:1722–26. 26412591 10.1016/j.injury.2015.06.015

[14] Sormaala MJ Niva MH Kiuru MJ. Outcomes of stress fractures of the talus. Am J Sports Med 2006;34:1809–14. 16902232 10.1177/0363546506291405

[15] Manzotti A Deromedis B Locatelli A. Intracancellous bone stress fracture of the talus in talo-calcaneal coalition: a case report. Foot and Ankle Surgery 1999;5:101–04.

[16] Motto SG. Stress fracture of the lateral process of the talus–a case report. Br J Sports Med 1993;27:275–76. 8130969 10.1136/bjsm.27.4.275 PMC1332020

[17] Apostu D Lucaciu O Oltean-Dan D. The influence of thyroid pathology on osteoporosis and fracture risk: a review. Diagnostics 2020;10:149. 32156092 10.3390/diagnostics10030149 PMC7151086

[18] Li X Zhang T Zhang H. Effects of levothyroxine therapy on bone and mineral metabolism in hypothyroidism: a systematic review and meta-analysis. BMC Endocr Disord 2025;25:11. 39810175 10.1186/s12902-024-01819-7 PMC11730139

[19] Paoletta M Moretti A Liguori S. Transient osteoporosis of the hip and subclinical hypothyroidism: an unusual dangerous duet? Case report and pathogenetic hypothesis. BMC Musculoskelet Disord 2020;21:543. 32791961 10.1186/s12891-020-03574-x PMC7427076

